# Prevalence and associated factors of depression, anxiety and stress among clinical therapists in China in the context of early COVID-19 pandemic

**DOI:** 10.3389/fpsyt.2024.1342528

**Published:** 2024-02-23

**Authors:** Rui Tao, Wenzheng Li, Kaiyuan Min, Daming Mo, Feng Geng, Lei Xia, Tingfang Liu, Yuanli Liu, Feng Jiang, Huanzhong Liu, Yi-lang Tang

**Affiliations:** ^1^ Department of Psychiatry, Chaohu Hospital of Anhui Medical University, Hefei, China; ^2^ Department of Psychiatry, School of Mental Health and Psychological Sciences, Anhui Medical University, Hefei, China; ^3^ Department of Psychiatry, Anhui Psychiatric Center, Hefei, China; ^4^ Department of Substance-Related Disorders, Hefei Fourth People’s Hospital, Hefei, China; ^5^ State Key Laboratory of Medical Molecular Biology, Institute of Basic Medical Sciences, Chinese Academy of Medical Sciences and Peking Union Medical College, Beijing, China; ^6^ Department of Psychiatry, Second Affiliated Hospital of Anhui Medical University, Hefei, China; ^7^ Research Department, School of Health Policy and Management, Chinese Academy of Medical Sciences & Peking Union Medical College, Beijing, China; ^8^ Research Department, School of International and Public Affairs, Shanghai Jiao Tong University, Shanghai, China; ^9^ Research Department, Institute of Healthy Yangtze River Delta, Shanghai Jiao Tong University, Shanghai, China; ^10^ Addiction Psychiatry Fellowship Program, Department of Psychiatry and Behavioral Sciences, Emory University, Atlanta, GA, United States; ^11^ Mental Health Service Line, Atlanta Veterans Affairs Medical Center, Decatur, GA, United States

**Keywords:** China, clinical therapists, depression, anxiety, stress, COVID-19 pandemic

## Abstract

**Objectives:**

To study the socio-demographic characteristics and the prevalence of depression, anxiety, and stress among clinical therapists in China during the early Coronavirus disease 2019 (COVID-19) pandemic and to identify associated factors.

**Method:**

This cross-sectional study was part of a multicenter, nationally representative survey conducted through WeChat from January 2021 to March 2021. Data, including socio-demographics, health-related behaviors, and information on whether they participated in the frontline work of treating COVID-19, were collected anonymously. Respondents also completed the Depression Anxiety Stress Scales-21 (DASS-21).

**Results:**

In total, 396 clinical therapists in the selected hospitals completed the questionnaires, with a response rate of 89.0%. Respondents were predominantly female (77.3%). About 6.6% of the participants were current tobacco users, and 20.7% had participated in the frontline work of treating COVID-19. Overall, 22.0%, 17.9%, and 8.8% of participants were classified as having clinically meaningful depression, anxiety, and stress, respectively, based on DASS-21 scores. Multiple logistic regression in Model 1 and Model 2 showed that depression, anxiety, and stress were associated with regular physical activity and frequent insomnia (all, p < 0.05). In anxiety model 2, the associated factors for anxiety during the pandemic were identified as education (master’s degree or more, OR=0.520; 95% CI=0.283-0.955), marital status (single, OR=2.064; 95% CI=1.022-4.168), tobacco use (OR=4.265; 95% CI=1.352-13.454), regular physical activity (OR=0.357; 95% CI=0.192-0.663), frequent insomnia (OR=6.298; 95% CI =2.522-15.729), and participation in the frontline work of treating COVID-19 (OR=3.179; 95% CI=1.697-5.954). The COVID-19 epidemic did not significantly increase the depression and stress levels among clinical therapists, but it did significantly increase anxiety levels.

**Conclusion:**

During the COVID-19 pandemic, depression, anxiety and stress were relatively common among clinical therapists in China. Regular physical activity and good sleep were important protective factors against emotional problems. Therefore, encouraging regular physical activity and actively addressing clinical therapists’ sleep problems is beneficial to improving the ability to cope with negative emotions. The COVID-19 epidemic significantly increased anxiety, and awareness and interventions should be recommended to reduce anxiety among clinical therapists during the COVID-19 pandemic.

## Introduction

The coronavirus disease 2019 (COVID-19) was caused by the severe acute respiratory syndrome coronavirus-2 (SARS-CoV-2), which was identified as a “pandemic” by the World Health Organization (WHO), had seriously affected the lives of people worldwide (1–4). As of 31 Dec 2021, 18.2 million deaths (as measured by excess mortality) have been estimated worldwide because of the COVID-19 pandemic over that period, of which 4820 deaths come from Mainland China (5). Due to the rapid spread and negative impacts on the physical and mental well-being of the global population, poor mental health outcomes are increasingly recognized among people in different parts of the world during the COVID-19 pandemic (6–9). Accumulating evidence suggests that COVID-19 can cause many mental illnesses in the general population, such as sleep disturbances (10, 11), depression (1, 12), anxiety (6, 13), stress (14) and suicidal behavior (15). A systematic review and meta-analysis estimated that the overall global prevalence of anxiety is 25% during the COVID-19 pandemic, which could be more than 3 times higher than normal (7.3%) in the general population (16).

The COVID-19 pandemic outbreak has also taken a substantial toll on the physical health and mental health of healthcare workers, and a high prevalence of mental health problems among healthcare workers has been reported and described all around the world (17–19), mainly including burnout (20, 21), posttraumatic stress symptoms (18, 22), depression, anxiety and stress (23, 24), and job satisfaction (25). Healthcare workers showed a meaningful worsening of stress, anxiety, and depression during the COVID-19 pandemic (26). Several studies have assessed the prevalence of depression (22.8%-34.4%), anxiety (20.7%-48.2%), and insomnia symptoms (37.9%-72.8%) among healthcare workers during the COVID-19 pandemic (21, 22, 26–29), which outstripped those of the general population during the pandemic (12, 22). A retrospective cohort study conducted in Mexico reported that depression (9%), stress (10%), and particularly anxiety (15%) increased in healthcare workers from the beginning to the COVID-19 pandemic peak (23). A cross-sectional web-survey study performed in Colombia reported that the prevalence of PTSD, anxiety, and depressive symptoms as 18.68%, 43.19%, and 26.85%, respectively. Additionally, these studies have also examined factors associated with depression, anxiety and stress in healthcare workers during the pandemic, which could be linked to working hours, burnout, sleeplessness and insomnia, substance use, worked on the ‘frontline’, and physical activity (19, 21, 24, 30–32).

To our knowledge, the presence of depression, anxiety, and stress can potentially alter the quality of care that healthcare workers are supposed to offer to their patients. Therefore, identifying depression, anxiety and stress among healthcare workers that would lead to prompt interventions is necessary. Clinical therapists are an emerging profession in China. According to the Chinese certification system and professional standards, the education and training backgrounds of clinical therapists are different, and they can be doctors, nurses, psychotherapists, and even social workers. As long as they have received formal training and obtained certification, these professionals are equipped to provide psychosocial interventions (33, 34). As an important part of healthcare workers, the role of Chinese clinical therapists is to provide timely therapeutic and preventive mental health care, which is essential in addressing the psychosocial needs of populations exposed to the COVID-19 pandemic. However, no prior studies have investigated the prevalence, socio-demographic characteristics, and depression, anxiety, and stress among clinical therapists in a national sample in China during the COVID-19 pandemic. Therefore, the objectives of this study were (1): to investigate the prevalence, socio-demographic characteristics, and depression, anxiety, and stress among Chinese clinical therapists during the COVID-19 pandemic based on a nationwide survey, and (2) to explore the relationship between participation in frontline work and mental health outcomes.

## Methods

### Study design, setting, and participants

This cross-sectional study was part of a multicenter, nationally representative survey conducted anonymously through WeChat from January 2021 to March 2021. We adopted whole-group sampling to investigate depression, anxiety and stress among clinical therapists and their associated factors during the early Covid-19 pandemic. 41 major tertiary psychiatric hospitals from 29 provinces were selected as targets and all clinical therapists in these hospitals were invited to participate in this survey. Before the study was made available to all participants, a pilot study was conducted in a small sample (n = 332, including doctors, nurses, and clinical therapists) to ensure high levels of internal consistency in the questionnaire (35), and we use the 10 events per variable (EPV) method to obtain a rough estimation of the study sample size (36). Finally, a total of 396 clinical therapists (n=445) were finally included in the statistical analyses, and the response rate was 89.0%. Socio-demographic variables (age, sex, educational level, and marital status), health-related behavior variables (tobacco use, alcohol use, regular physical activity, and frequent insomnia), and information on whether clinical therapists had participated in the frontline work of treating COVID-19 were collected with the online questionnaire. The Depression Anxiety Stress Scales-21 (DASS-21) was used to measure the emotional state of depression, anxiety, and stress. Due to the safety and convenience of WeChat platform, we decided to use WeChat as the primary tool for investigating the mental health of clinical therapists, and the questionnaire was administered in Chinese.

The research protocol was approved by the Ethics Committee of Chaohu Hospital of Anhui Medical University and an electronic consent form was obtained from each participant.

### Questionnaire

We developed the electronic questionnaire based on literature reviews and expert opinions. DASS-21 is a self-report instrument specifically designed to measure the emotional state of depression, anxiety, and stress (37), which has been recognized by researchers in different countries (38–40). DASS-21 is a concise version of the DASS-42 that has been demonstrated to have similar accuracy to the full DASS. For DASS-21, there are seven items in each of the subscales (depression, anxiety, and stress), and each item is scored on a 4-grade Likert scale from 0 (did not apply to me at all) to 3 (applied to me very much or most of the time). The highest possible score is 42 for each subscale, and participants are classified as “clinically meaningful” when their scores are ≥ 10 for depression, ≥ 8 for anxiety and ≥ 15 for stress, respectively. Higher scores on each subscale indicate higher levels of depression, anxiety and stress.

### Statistical analyses

The sample distribution was conducted using mean ± standard deviation for continuous variables and numbers and percentages for categorical variables. For the statistical analyses, the Chi-square test was utilized to assess the categorical variables and other variables that were not in the normal distribution. The prevalence of clinically meaningful depression (≥ 10), anxiety (≥ 8), and stress (≥ 15) were computed and compared across sociodemographic variables and other variables, including socio-demographic variables (age, sex - male and female, educational level - bachelor’s degree or less and master’s degree or more, and marital status - single, married, divorced/widowed), health-related behavior variables (tobacco use - yes/no), alcohol use - yes/no, regular physical activity - yes/no, and frequent insomnia - yes/no), and participation in the frontline work of treating COVID-19. Three 2-model multiple logistic regressions were conducted to test the association of socio-demographic variables, health-related behavior variables, and participation in the frontline work of treating COVID-19 with anxiety, depression, and stress. Model 1 consisted of socio-demographic variables and health-related behavior variables. Model 2 included Model 1’s variables and a covariate variable (participation in the frontline work of treating COVID-19). All statistical analyses were performed using SPSS version 22.0 at the 0.05 significance level (two-tailed).

## Results

### Socio-demographic characteristics of clinical therapists in china

The socio-demographic characteristics of those participants are displayed in **Table 1**. A total of 396 participants were included in this study, aged 23 to 59 (33.87 ± 7.62) years. Nearly 77.3% (306/396) of the participants were female, 39.1% (155/396) were a master’s degree or more, and 62.1% (246/396) were married. About 6.6% (26/396) of the participants were current tobacco users, 39.1% (155/396) were current alcohol users, 75.5% (299/396) of participants reported regularly exercising, and 72% (285/396) experienced frequent insomnia. Of all the participants, 20.7% (82/396) had participated in the frontline work of treating COVID-19.

**Table 1:** Differences in depression, anxiety, and stress by demographic characteristics (n=396).

Variables	Participants	Depression		Anxiety		Stress	
	n=396	n=87	Z/χ^2^	n=71	Z/χ^2^	n=35	Z/χ^2^
Age (years) (Mean ± SD)	33.87±7.62	33.55±6.75	-0.248	33.34±7.20	-0.379	32.11±7.46	-1.606
Sex (N, %)							
Male	90 (22.7)	20 (23.0)		16 (22.5)		11 (31.4)	
Female	306 (77.3)	67 (77.0)	0.004	55 (77.5)	0.002	24 (68.6)	1.655
Education (N, %)							
Bachelor's degree or less	241 (60.9)	62 (71.3)		51 (71.8)		26 (74.3)	
Master degree's or more	155 (39.1)	25 (28.7)	5.068^*^	20 (28.2)	4.372^*^	9 (25.7)	2.906
Marital status (N, %)							
Married	246 (62.1)	52 (59.8)		38 (53.5)		16 (45.7)	
Single	135 (34.1)	31 (35.6)		29 (40.8)		17 (48.6)	
Divorced or widowed	15 (3.8)	4 (4.6)	0.370	4 (5.6)	2.966	2 (5.7)	4.401
Tobacco use (N, %)							
No	370 (93.4)	79 (90.8)		63 (88.7)		31 (88.6)	
Yes	26 (6.6)	8 (9.2)	1.257	8 (11.3)	3.118	4 (11.4)	1.480
Alcohol use (N, %)							
No	241 (60.9)	48 (55.2)		40 (56.3)		22 (62.9)	
Yes	155 (39.1)	39 (44.8)	1.513	31 (43.7)	0.742	13 (37.1)	0.064
Physical activity (N, %)							
No	97 (24.5)	33 (37.9)		27 (38.0)		14 (40.0)	
Yes	299 (75.5)	54 (62.1)	10.883^**^	44 (62.0)	8.567^**^	21 (60.0)	4.991^*^
Frequent insomnia (N, %)							
No	111 (28.0)	10 (11.5)		6 (8.5)		2 (5.7)	
Yes	285 (72.0)	77 (88.5)	15.113^***^	65 (91.5)	16.440^***^	33 (94.3)	9.478^**^
In the frontline work of COVID-19 (N, %)							
No	314 (79.3)	67 (77.0)		47 (66.2)		25 (71.4)	
Yes	82 (20.7)	20 (23.0)	0.353	24 (33.8)	9.036^**^	10 (28.6)	1.446

^*^ p<0.05. ^**^ p<0.01. ^***^ p<0.001.

Some common variables were significantly associated with depression, anxiety and stress among clinical therapists, such as regular physical activity and frequent insomnia (all, *p* < 0.05). Less physical activity and frequent insomnia were more commonly seen among the clinical therapists who suffered from depression, anxiety and stress. No sex or alcohol use differences were found in the prevalence of depression, anxiety and stress.

### Prevalence of depression, anxiety and stress

**Table 2** shows the prevalence of anxiety, depression, and stress among clinical therapists. Overall, 22.0%, 17.9%, and 8.8% of participants were classified as having clinically meaningful depression, anxiety, and stress, respectively. Of all participants in this study, 11.8% (47/396) reported moderate to extremely severe depression, 12.3% (49/396) reported moderate to extremely severe anxiety and 3.8% (15/396) reported moderate to extremely severe stress. The prevalence of moderate to extremely severe depression, anxiety and stress in females was 9.5% (29/306), 12.1% (37/306) and 3.9% (12/306), respectively. 33.8% (24/71) of those with clinically meaningful anxiety had participated in the frontline work of treating COVID-19 in the past year.

**Table 2:** The prevalence of depression, anxiety and stress among the participants [n (%), Mean±SD ].

Variable	Normal	Mild	Moderate	Severe and extremely severe
Depression	309 (78.0), 0 (0, 4)	40 (10.1), 10.55±0.90	33 (8.3), 15.15±1.66	14 (3.5), 29.43±5.94
Anxiety	325 (82.1), 2 (0, 4)	22 (5.6), 8.00±1.05	37 (9.3), 11.83±1.66	12 (3.0), 20.67±5.35
Stress	361 (91.2), 4 (0, 8)	20 (5.1), 16.70±0.98	8 (2.0), 21.25±1.04	7 (1.8), 39.14±3.43

### Factors associated with depression

**Table 3** displays the references of the categorical variables and the association between socio-demographic characteristics, lifestyle, and participation in the frontline work of treating COVID-19 and depression. In Model 1, we performed multiple logistic regression analyses with depression as the independent variable and socio-demographic characteristics and lifestyle as the dependent variables (Nagelkerke R^2 = 0.139, Hosmer-Lemeshow: *p*=0.332). Depression was associated with education (master’s degree or more, OR =0.549; 95% CI =0.318-0.949), regular physical activity (OR =0.369; 95% CI =0.211-0.644), and frequent insomnia (OR =3.834; 95% CI =1.876-7.834). Higher education and regular physical activity were protective factors for depression. In Model 2, we added the variable of participation in the frontline work of treating COVID-19 as a covariate in multiple logistic regression analyses (Nagelkerke R^2 = 0.142, Hosmer-Lemeshow: *p*=0.448), and the depression model was not significantly altered by the addition of the variable of participation in the frontline work of treating COVID-19.

**Table 3:** Multiple logistic regression of demographic characteristics with depression in clinical therapists.

Variables	Model 1					Model 2				
	Coef.	SE	*p*	OR	95%CI	Coef.	SE	*p*	OR	95%CI
Age (years)	-0.008	0.021	0.705	0.992	0.952-1.034	-0.007	0.021	0.728	0.993	0.953-1.034
Female (ref. Male)	0.373	0.365	0.306	1.452	0.711-2.969	0.387	0.365	0.289	1.472	0.953-10.34
Education (ref. Bachelor's degree)										
Master degree's or more	-0.599	0.278	0.031^*^	0.549	0.318-0.947	0.604	0.278	0.030^*^	0.547	0.317-0.943
Marital status (ref. Married)										
Single	0.191	0.321	0.552	1.211	0.645-2.271	0.213	0.322	0.508	1.238	0.658-2.328
Divorced or widowed	0.225	0.676	0.739	1.252	0.333-4.713	0.238	0.678	0.726	1.269	0.336-4.793
Tobacco use (ref. No)	0.686	0.540	0.204	1.986	0.689-5.722	0.723	0.541	0.182	2.060	0.713-5.951
Alcohol use (ref. No)	0.410	0.285	0.151	1.507	0.861-2.638	0.404	0.286	0.157	1.498	0.856-2.623
Physical activity (ref. No)	-0.998	0.285	<0.001^***^	0.369	0.211-0.644	-1.006	0.285	<0.001^***^	0.366	0.209-0.640
Frequent insomnia (ref. No)	1.344	0.365	<0.001^***^	3.834	1.876-7.834	1.358	0.366	<0.001^***^	3.888	1.898-7.965
In the frontline work (ref. No)						0.278	0.308	0.367	1.321	0.722-2.417

Coef., coefficient; SE, standard error; p, p-value; OR, Odds Ratio; 95%CI, 95% Confidence Interval; ref., reference.

References: Sex (Male), Education (Bachelor's degree or less), Marital status (Married), Tobacco use (No), Alcohol use (No), Regular exercise (No), Frequent insomnia (No) and participated the frontline work of COVID-19 (No). ^*^ p<0.05. ^**^ p<0.01. ^***^ p<0.001.

### Factors associated with anxiety

**Table 4** shows the association between anxiety and socio-demographic characteristics, lifestyle, and participation in the frontline work of treating COVID-19 by multiple logistic regression analyses. In model 1, education (master’s degree or more), tobacco use, regular physical activity and frequent insomnia were associated with anxiety among clinical therapists (Nagelkerke R^2 = 0.166, Hosmer-Lemeshow: *p*=0.715). In model 2, the inclusion of participation in the frontline work of treating COVID-19 significantly increased the explained variance for anxiety model 2 (Nagelkerke R^2 = 0.213, Hosmer-Lemeshow: *p*=0.270). The results showed that anxiety was associated with education (master’s degree or more, OR=0.520; 95% CI=0.283-0.955), marital status (single, OR=2.064; 95% CI=1.022-4.168), tobacco use (OR=4.265; 95% CI=1.352-13.454), regular physical activity (OR =0.357; 95% CI =0.192-0.663), frequent insomnia (OR =6.298; 95% CI=2.522-15.729) and participation in the frontline work of treating COVID-19 (OR=3.179; 95% CI=1.697-5.954). However, a negative association was observed between depression and anxiety and higher education levels than a bachelor’s degree or less (**Tables 3**, **4**).

**Table 4:** Multiple logistic regression of demographic characteristics with anxiety in clinical therapists.

Variables	Model 1					Model 2				
	Coef.	SE	*p*	OR	95%CI	Coef.	SE	*p*	OR	95%CI
Age (years)	-0.006	0.023	0.800	0.994	0.950-1.040	-0.003	0.024	0.888	0.997	0.951-1.045
Female (ref. Male)	0.602	0.412	0.143	1.827	0.815-4.093	0.674	0.418	0.107	1.962	0.864-4.454
Education (ref. Bachelor's degree)										
Master degree's or more	-0.627	0.305	0.040^*^	0.534	0.294-0.971	-0.653	0.310	0.035^*^	0.520	0.283-0.955
Marital status (ref. Married)										
Single	0.596	0.349	0.087	1.815	0.916-3.595	0.725	0.359	0.043^*^	2.064	1.022-4.168
Divorced or widowed	0.713	0.687	0.299	2.041	0.531-7.837	0.829	0.703	0.238	2.291	0.578-9.085
Tobacco use (ref. No)	1.254	0.570	0.028^*^	3.505	1.146-10.719	1.451	0.586	0.013^*^	4.265	1.352-13.454
Alcohol use (ref. No)	0.252	0.311	0.418	1.286	0.700-2.365	0.228	0.317	0.472	1.256	0.675-2.339
Physical activity (ref. No)	-0.964	0.308	0.002^**^	0.382	0.209-0.698	-1.030	0.316	0.001^**^	0.357	0.192-0.663
Frequent insomnia (ref. No)	1.705	0.451	<0.001^***^	5.500	2.272-13.313	1.840	0.467	<0.001^***^	6.298	2.522-15.729
In the frontline work (ref. No)						1.157	0.320	<0.001^***^	3.179	1.697-5.954

### Factors associated with stress

As shown in **Table 5**, there was a significant association between regular physical activity, frequent insomnia and stress in Model 1 (Nagelkerke R^2 = 0.152, Hosmer-Lemeshow: *p*=0.391) and Model 2 (Nagelkerke R^2 = 0.166, Hosmer-Lemeshow: *p*=0.353). Regular physical activity and good sleep were protective factors against depression, anxiety, and stress among clinical therapists. The COVID-19 epidemic did not significantly increase the clinical therapists’ levels of depression and stress, but it did significantly increase anxiety levels.

**Table 5:** Multiple logistic regression of demographic characteristics with stress in clinical therapists.

Variables	Model 1					Model 2				
	Coef.	SE	*p*	OR	95%CI	Coef.	SE	*p*	OR	95%CI
Age (years)	-0.026	0.033	0.427	0.974	0.912-1.040	-0.026	0.034	0.449	0.975	0.912-1.042
Female (ref. Male)	-0.252	0.493	0.609	0.777	0.296-2.042	-0.202	0.495	0.684	0.817	0.310-2.155
Education (ref. Bachelor's degree)										
Master degree's or more	-0.627	0.419	0.134	0.534	0.235-1.213	-0.630	0.419	0.133	0.532	0.234-1.211
Marital status (ref. Married)										
Single	0.674	0.456	0.139	1.963	0.804-4.793	0.748	0.462	0.105	2.113	0.855-5.223
Divorced or widowed	1.104	0.879	0.209	3.017	0.539-16.887	1.181	0.899	0.189	3.257	0.559-18.975
Tobacco use (ref. No)	0.809	0.711	0.255	2.245	0.557-9.042	0.932	0.716	0.193	2.540	0.624-10.340
Alcohol use (ref. No)	-0.345	0.425	0.417	0.708	0.308-1.629	-0.364	0.428	0.395	0.695	0.301-1.607
Physical activity (ref. No)	-0.797	0.394	0.043^*^	0.451	0.208-0.976	-0.824	0.398	0.039^*^	0.439	0.201-0.957
Frequent insomnia (ref. No)	2.016	0.744	0.007^**^	7.510	1.749-32.254	2.099	0.751	0.005^**^	8.159	1.872-35.557
In the frontline work (ref. No)						0.707	0.425	0.096	2.029	0.883-4.662

## Discussion

This was the first national survey to investigate the prevalence of depression, anxiety and stress and their correlates among Chinese clinical therapists during the COVID-19 pandemic. As an important part of healthcare workers, clinical therapists are different from doctors and nurses because of their professional characteristics. Their professional characteristics are particularly beneficial in dealing with negative emotions at work and in personal lives. Exploring the professional characteristics of clinical therapists is beneficial for utilizing these traits to assist other healthcare professionals in coping with emotional issues encountered in their work. In our study, we found that depression, anxiety and stress were relatively common among Chinese clinical therapists, with 22.0% reporting clinically meaningful depression, 17.9% reporting clinically meaningful anxiety, and 8.8% reporting clinically meaningful stress, respectively. In addition, the prevalence of clinically meaningful depression, anxiety and stress symptoms during the pandemic was significantly higher among those who were female and had a low educational level (bachelor’s degree or less). Multivariable logistic regression also revealed that the covariates associated with significant depression, anxiety, and stress symptoms during the pandemic were physical activity and frequent insomnia. These prevalences are comparable to those in a population-based, cross-sectional study conducted in Iran (41), which found the prevalence of depression, anxiety, and stress to be 4.79%, 13.28%, and 15.13%, respectively. In a nationally representative cross-sectional study conducted in Korea, the prevalence of significant depressive symptoms in younger adults during the pandemic was 7.4%, which was higher than in the pre-pandemic group (4.7%) (12). At the same time, a rapid systematic review and meta-analysis reported that the prevalence of depression, anxiety and stress among health professionals was 37.12% (95% CI: 31.80–42.43), 41.42% (95% CI: 36.17–46.54) and 44.86% (95% CI: 36.98–52.74) during the COVID-19 pandemic (42).

Somewhat interestingly, we found that working on the frontline of treating COVID-19 was not significantly associated with either depression or stress, but it did increase the level of anxiety among clinical therapists according to our study. One potential explanation is that clinical therapists’ professional advantages and good health-related habits (such as physical activity and healthy sleep) allow them to better cope with negative emotions (43–45). Psychotechnical training and professional supervision of clinical therapists enables them to deal with negative emotions in themselves and visitors in different situations (33). Another potential explanation is the selective dynamic temporal interplay between anxiety about the COVID-19 pandemic and negative emotional states. A network analysis study of 1145 adults living in the Netherlands and Belgium found that increased COVID-19 related anxiety revealed temporal associations that may impact the dynamic regulation of emotional states over a longer time (6). This survey was conducted shortly after the COVID-19 pandemic outbreak, which acted as a major stressor and significantly increased anxiety among clinical therapists. This finding is consistent with a previous study reporting that anxiety symptoms increased immediately after the onset of the COVID-19 pandemic (7) and presented a significant decrease at six months follow-up (30). In contrast, the effects of the COVID-19 pandemic on depression and stress may have taken longer to manifest. Nonetheless, anxiety remains an important issue for clinical therapists during the early stages of the COVID-19 pandemic.

### Socio-demographics characteristics and depression, anxiety and stress

Our findings obtained from this study indicated that a higher education level (Master’s degree or more) was negatively associated with depression and anxiety. This finding is in line with the results of previous studies conducted on U.S. adults (46), which showed that lower education levels are more vulnerable to depression and anxiety. Seemingly, it has been found that having high information about the COVID-19 pandemic and a more comprehensive knowledge structure in mind and kinds of skills can enhance individuals’ ability to cope and help alleviate negative emotions in a timely manner (47). However, this finding contradicts the results reported from another country. In Afghanistan, no significant association was found between educational level and the mental illnesses under study (48). It is possible that cultural or contextual factors may contribute to these differences.

Additionally, another finding of this study showed that sex and marital status were not directly related to depression and stress. In the context of the epidemic, single clinical therapists were more likely to experience more anxiety, which was not in line with the findings of some previous studies. These studies suggested a higher incidence of depression and stress in single or divorced individuals, as they could not draw support from their family (49) and experienced more loneliness (50, 51). It is argued that the potential professional advantages that allow them to be better equipped to cope with negative emotions played an important part among clinical therapists (45).

### Health-related behaviors and depression, anxiety and stress

The results of the present study demonstrated that less physical activity and frequent insomnia were more commonly seen among the clinical therapists who suffered from depression, anxiety, and stress. Previous studies have documented the relationship between regular physical activity (52, 53), frequent insomnia (10, 11) and mental health. Physical activity is widely recognized as a protective factor against mental health problems (30, 54, 55). This study identified that 75.5% of participants reported engaging in physical activity regularly. Previous studies have documented that moderate physical activity is associated with lower depression, anxiety and stress in healthcare workers during the COVID-19 pandemic (23). Physical activity also had a positive effect on the perception of stress (54) and decreased physical activity was also associated with increased depression symptom (56). Physical activity at any level could buffer against the effects of work intensity and alleviate the effect of working hours or working days on depression symptoms, which might offer a helpful strategy for improving mental health problems in different populations (55, 57). For example, one study on the relationship between physical activity and mental health after the onset of the COVID-19 found that physical activity by 52.5 min of moderate-intensity physical activity per week effectively reduced mental distress (52). Our data indicate that physical activity may aid in the control of the burden of mental health problems as a consequence of the COVID-19 pandemic in China, and people who experience high levels of depression, anxiety and stress due to the pandemic may benefit from regular physical activity (55, 58).

The study also identified that the prevalence of frequent insomnia in clinical therapists during the COVID-19 pandemic was 72.0%, which was higher than the rates of 20%-39.1% reported in other populations (11, 28, 59). Evidence suggests that insomnia symptoms are highly prevalent among healthcare workers during the COVID-19 pandemic (32), and participants with frequent insomnia had a greater risk of subsequent mental health problems (60). Insomnia symptoms share a bidirectional relationship with mental disorders and contribute to the development and maintenance of negative emotional symptoms (10, 61). A longitudinal study reported that during the first year of the pandemic, depression symptoms predicted subsequent insomnia symptoms one year later, and conversely that insomnia symptoms predicted depression symptoms (61). Previous studies suggested the pandemic’s mixed but potentially negative impact on people’s sleep health (62), and individuals with insomnia symptoms were more likely to experience mental health problems after the pandemic outbreak (10, 11). A longitudinal study of Canadian community adults (63) and a cross-sectional study of the Chinese general public (14) found that insomnia was an independent predictor for the clinically meaningful in overall posttraumatic stress disorder symptoms and the severity of emotional symptoms, and individuals with insomnia symptoms (stable-high and increasing) reported significantly higher levels of depression during the COVID-19 pandemic (11). Hom MA., et al. (2016) have proposed that good sleep could promote individuals to efficiently deploy their cognition-related resources and increase the likelihood of utilizing emotion regulation strategies to regulate emotional experiences (64).

In this study, we found that the prevalence of tobacco use among clinical therapists was 6.6%, which was lower than that reported among mental health workers in China (8.6%) (65) and a significant association between tobacco use and anxiety. Based on the results of the logistic analysis (**Table 4**, anxiety model 2), current cigarette smokers were four times more likely to be suffering from anxiety symptoms (OR= 4.265; 95%CI=1.352–13.454). One possible explanation is that cigarettes effectively alleviate anxiety symptoms among frontline clinical therapists (66, 67).

### Participated in the frontline work of treating COVID-19 and depression, anxiety and stress

The COVID-19 pandemic has necessitated healthcare workers operating on the frontlines. Most studies have reported a higher frequency of mental health problems in frontline healthcare workers attending COVID-19 patients. The mental health of “frontline” workers was affected in complex and multifaceted ways by the COVID-19 epidemic (31). Evidence suggests that anxiety symptoms increased worldwide due to the COVID-19 pandemic (68). In our study, 20.7% had participated in the frontline work of treating COVID-19, and clinical therapists who participated in the frontline work of treating COVID-19 only presented a significant increase in anxiety proportion. This finding is consistent with a previous study reporting that healthcare workers, only those assigned to COVID-19 areas presented a statistically significant increase in anxiety symptoms, which could be attributed to worries about contamination, moral injury, and work stress (23). In the early stage of the outbreak, anxiety among clinical therapists significantly increased due to worries and a lack of understanding of the COVID-19 epidemic (6, 7).

## Limitations

However, three limitations in our study should be considered when interpreting these results. The first limitation of the present research study is that we collected data based on self-reports of the prevalence of depression, anxiety and stress, and we did not use clinical measures (such as DSM-5). Second, as is the case in most cross-sectional surveys, our results were un able to infer the causal relationship between depression, anxiety and stress and their associated factors. Third, this study was an anonymous survey, which did not allow for targeted interventions for participants with depression, anxiety and stress identified in the survey. It is noteworthy that as a nation-wide survey, the sample size of clinical therapists was only 445, which should be targeted and supplemented in terms of medical resources for public mental health.

## Conclusions

To conclude, based on this survey of a nationwide sample of clinical therapists from 41 tertiary psychiatric hospitals across China, we found that the overall prevalence of depression (22.0%), anxiety (17.9%), and stress (8.8%) was relatively common among clinical therapists in China during the COVID-19 pandemic. Additionally, maintaining a certain healthy lifestyle is important for mental health, as regular physical activity and healthy sleep are important protective factors for emotional problems. Therefore, encouraging regular physical activity and actively addressing clinical therapists’ sleep problems are beneficial to improving their ability to cope with negative emotions. The COVID-19 epidemic significantly increased anxiety, and it is recommended to raise awareness and provide interventions to reduce anxiety among clinical therapists during the COVID-19 pandemic.

## Data availability statement

The raw data supporting the conclusions of this article will be made available by the authors, without undue reservation.

## Ethics statement

The research protocol was approved by the Ethics Committee of Chaohu Hospital of Anhui Medical University and an electronic consent form was obtained from each participant.

## Author contributions

RT: Writing – original draft, Writing – review & editing. WL: Investigation, Writing – review & editing. KM: Formal analysis, Writing – review & editing. DM: Investigation, Writing – review & editing. FG: Investigation, Writing – review & editing. LX: Investigation, Writing – review & editing. TL: Resources, Writing – review & editing. YL: Resources, Writing – review & editing. FJ: Project administration, Writing – review & editing. HL: Funding acquisition, Project administration, Supervision, Writing – review & editing. Y-LT: Data curation, Methodology, Supervision, Writing – review & editing.
